# Lung adenocarcinoma with peculiar growth to the pulmonary artery and thrombus formation: report of a case

**DOI:** 10.1186/1477-7819-10-16

**Published:** 2012-01-21

**Authors:** Taichiro Goto, Arafumi Maeshima, Ryoichi Kato

**Affiliations:** 1 Department of General Thoracic Surgery, National Hospital Organization Tokyo Medical Center, Tokyo, Japan; 2 Department of Pathology, National Hospital Organization Tokyo Medical Center, Tokyo, Japan

## Abstract

**Background:**

Cases of pulmonary artery masses have only rarely been reported, and the optimal type of the diagnosis and treatment is controversial.

**Case Presentation:**

An 81-year-old woman was found to have an abnormal shadow on chest X-ray film. Computed tomography showed an irregularly bordered tumor centered in the hilar region extending from segment 6 to the middle lobe of the right lung. Pulmonary angiography showed complete occlusion of the trunk at the periphery proximal to the bifurcation of the posterior ascending branch. Based on bronchoscopic biopsy of the tumor, an adenocarcinoma was diagnosed. Middle and lower lobectomy was performed. Histopathologically, the adenocarcinoma had invaded the tunica intima of the pulmonary artery and also replaced the endothelium in the same region. Although a large thrombus was found at the vessel invasion site of the adenocarcinoma in the pulmonary artery, there were no malignant findings in the thrombus itself.

**Conclusions:**

This is the first reported case of radical resection of a lung cancer with invasion along the pulmonary artery wherein a benign thrombus had formed. In general, surgery would be the treatment of choice for a pulmonary artery mass.

## Background

Tumors growing into the pulmonary arteries include angiosarcoma and tumor embolizations from other organs [1-5]. Despite being rare, there are several reports of primary lung cancers macroscopically growing into pulmonary arteries [6-8]. An intravascular tumor at the pulmonary artery represents a rare but important differential diagnosis of pulmonary thromboembolism [1,5,9]. Herein, we describe a patient with lung cancer that presented as a suspected pulmonary artery mass. The evaluation and treatment of this patient are presented in detail.

## Case Presentation

**Figure 1:**
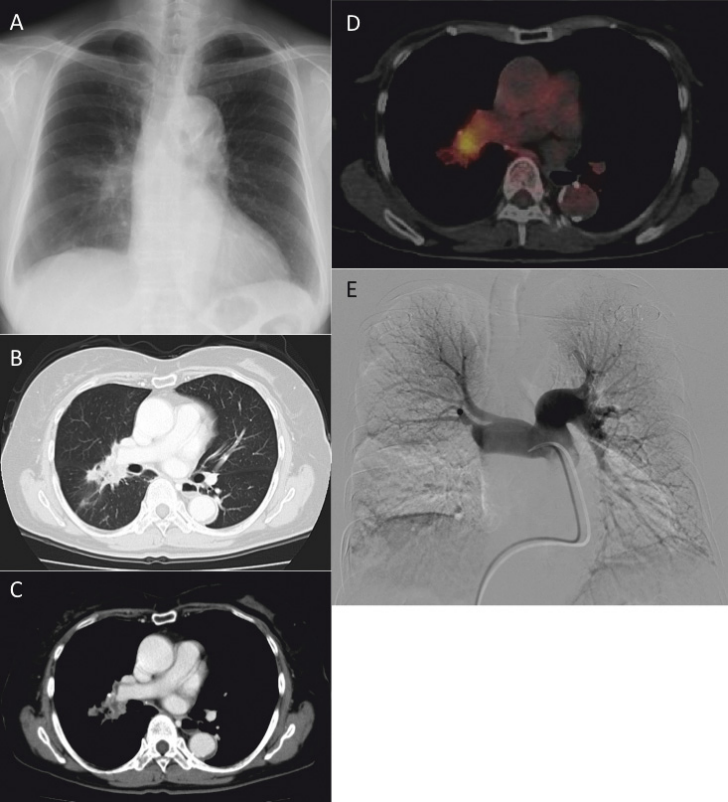
**Radiological findings**. A: Chest X-ray showed a mass shadow in the right middle lung field. B, C: Chest CT showed a tumor with an irregular border centered in the hilar region extending from S6 to the middle lobe of the right lung. Stenosis and occlusion of the pulmonary artery were found on mediastinal window setting. D: Positron emission tomography showed fluorodeoxyglucose uptake in the tumor. E: Angiography showed complete occlusion of the pulmonary trunk at the periphery proximal to the bifurcation of the posterior ascending branch, and a filling defect at the root of this branch.

An 81-year-old woman was found to have an abnormal shadow in the right lower lung field on chest radiography for a routine health check-up (Figure 1A). Her past medical history was unremarkable. Her smoking history was 1 pack/day × 50 years, and she had quit smoking at age 70. Chest computed tomography (CT) showed a tumor with an irregular border centered in the hilar region extending from segment 6 (S6) to the middle lobe of the right lung (Figure 1B, C). Based on stenotic and occlusive findings of the pulmonary artery on CT scan, the tumor was considered to have directly invaded the pulmonary trunk (Figure 1B, C). Although positron emission tomography showed fluorodeoxyglucose uptake with a maximum standard uptake value of 4.7 in the tumor region (Figure 1D), there was no fluorodeoxyglucose uptake in mediastinal lymph nodes or other organs. To closely examine the extent of proximal intravascular tumor invasion, angiography was performed, which revealed complete occlusion of the pulmonary trunk at the periphery proximal to the bifurcation of the posterior ascending branch, and a filling defect at the root of this branch (Figure 1E). Although bronchoscopy showed no mass lesion in the visible range, adenocarcinoma was diagnosed by bronchoscopic biopsy of the tumor. The level of carcinoembryonic antigen was elevated, at 5.7 ng/mL (institutional cutoff value, 5.0 ng/ml); however, no abnormalities were detected in other blood chemistry or tumor marker levels. The patient was otherwise healthy and asymptomatic. Her vital capacity was 1.92 liters, and her forced expiratory volume in 1 second was 1.40 liters. Because right pneumonectomy would be difficult given her pulmonary function and age, preservation of the right upper lobe was chosen as the surgical strategy.

**Figure 2:**
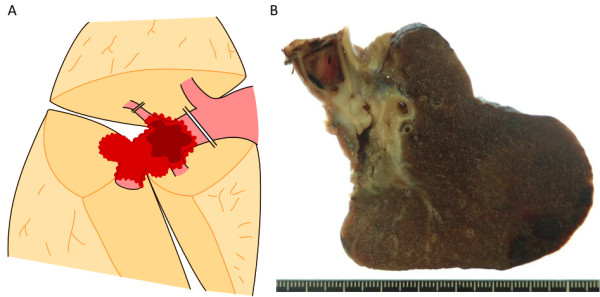
**Surgical findings**. A: Intraoperative treatment for blood vessels. The tumor invaded the pulmonary trunk on the interlobar surface and also at the root of the posterior ascending branch. The branch was ligated and divided on the peripheral side. The pulmonary trunk was clamped with vascular forceps at the periphery proximal to the bifurcation of the anterior trunk, and the pulmonary trunk was then divided and sutured. The red area indicates the cancer invasion site. The brown area indicates the thrombus in the pulmonary artery. The double lines indicate the division sites of blood vessels. B: Macroscopically, the cancer showed continuous invasion of the pulmonary artery, and the pulmonary trunk was occluded with a massive thrombus.

Intraoperatively, the tumor was found to be located on the interlobar surface of S6-middle lobe, tending to invade proximally and distally along the pulmonary trunk. Massive induration was felt inside the pulmonary artery at the cancer invasion site (Figure 2A). Cancer invasion was also observed at the root of the posterior ascending branch, and induration was felt in the lumen of this branch. Thus, it was ligated and divided on the peripheral side (Figure 2A). The pulmonary artery was clamped with vascular forceps at a site proximally peripheral to the bifurcation of the anterior trunk and without internal palpable induration; then, the artery was divided and sutured. As a result of these procedures on blood vessels, we were able to preserve the right upper lobe and perform middle and lower lobectomy (Figure 2A).

**Figure 3:**
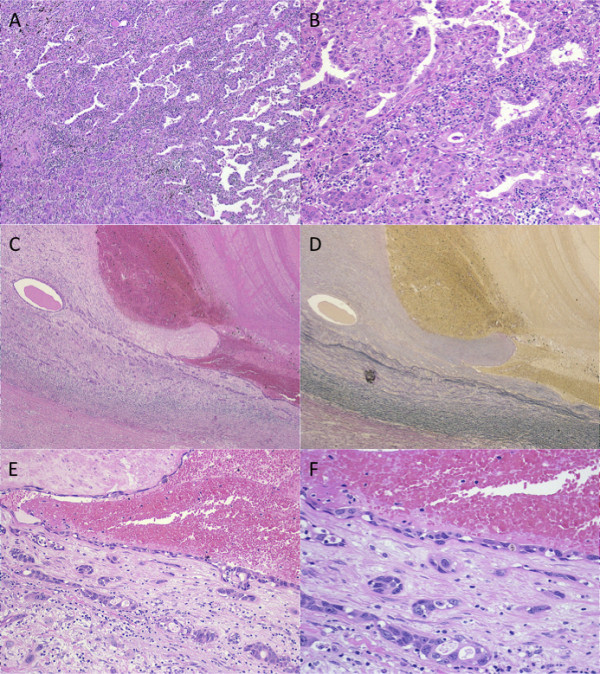
**Histological findings**. A, B: The center of the adenocarcinoma is mainly composed of bronchioloalveolar, solid and acinar pattern. C, D: Adenocarcinoma invasion was observed in the pulmonary artery wall. Thrombus formation was seen in the blood vessel (C, Hematoxylin and eosin staining; D, Elastica-van Gieson staining). E, F: Adenocarcinoma mainly composed of acinar pattern invaded the tunica intima of the pulmonary artery. The endothelium of the same region had been replaced by adenocarcinoma cells.

Gross examination of the specimen disclosed that the tumor was mainly centered in the hilar region at S6, measured 40 mm, and was partially grayish inside (Figure 2B). The tumor showed continuous invasion into the pulmonary artery, and the pulmonary trunk was occluded with a massive thrombus. Histopathologically, cuboidal tumor cells showed acinar, bronchioloalveolar and solid pattern with central fibrotic scar, leading to the diagnosis of adenocarcinoma with mixed subtypes (Figure 3A, B). Invasion of adenocarcinoma mainly composed of acinar pattern was observed in the pulmonary artery wall (Figure 3C, D), and the endothelium had also been replaced by adenocarcinoma cells in the same region (Figure 3E, F). Although a large thrombus was detected at the site of vessel invasion by adenocarcinoma in the pulmonary artery, there were no malignant findings in the thrombus itself (Figure 3C-F). No cancer invasion was observed at the surgical margins of either the posterior ascending branch or the pulmonary trunk (Figure 2A). There was no lymph node metastasis. The cancer was pathologically diagnosed as pT4N0M0, stage IIIA.

The patient's postoperative course was uneventful, and she has remained in good health since discharge. In consideration of her advanced age, no adjuvant chemotherapy was performed. For the 14 months to date, since surgery, the patient has remained free of lung cancer recurrence, and will continue to be followed-up on an outpatient basis.

## Discussion

We experienced a case with lung cancer directly invading the pulmonary artery and forming a thrombus at the vessel invasion site. To the best of our knowledge, no similar cases have been reported previously. The primary etiologies of pulmonary artery masses include pulmonary thromboembolism, angiosarcoma, tumor embolism from cancers of other organs, and growth of a primary lung cancer into the intravascular lumen [1-9], but the diagnosis of a pulmonary mass is seldom made preoperatively. There are very few reports describing primary lung cancers directly invading pulmonary arteries, with polypoidal growth into the lumens of these blood vessels [6-8]. Although the pathology of our case may also be regarded as direct invasion of a primary lung cancer to the pulmonary artery, this case featured temporal and spatial coincidence of vessel invasion by the lung cancer and a thrombus without malignant findings. The well-known pathologies of cancer-related thrombus include Trousseau's syndrome, pulmonary tumor thrombotic microangiopathy, and disseminated intravascular coagulation, which are multiple microthrombi associated with biochemical changes caused by vessel invasion of cancer [10,11]. On the other hand, an intravascular massive thrombus was formed at the vessel invasion site of the cancer in our present case. One possible explanation is that peripheral pulmonary arterial occlusion by the tumor disturbed arterial flow at a proximal site, leading to the formation of a thrombus, but the precise pathogenesis remains uncertain. Studies of similar cases and further definition of this condition may shed light on the complex underlying mechanisms.

The rarity of this tumor growth pattern makes it difficult to define its prognostic impact and pathological implications. For example, polypoidal cancer growth in a pulmonary artery is thought to lead to dissemination into the pulmonary circulation. However, such pathological findings are actually quite infrequent. Pulmonary artery invasion reportedly carries a better prognosis than other forms of mediastinal structural invasion [12]. Thus, even when macroscopic cancer invasion to the main pulmonary artery seems likely, complete surgical resection may offer the only chance for survival.

## Conclusions

In conclusion, this is the first reported case of a lung cancer mimicking pulmonary artery sarcoma, but which actually formed a massive thrombus at the vessel invasion site. This mode of cancer extension is quite rare and its prognostic significance remains unclear. Although pulmonary arterial invasion is generally an ominous prognostic sign, presentation as a pulmonary artery mass may warrant open exploration for diagnosis and possible definitive treatment.

## Consent

Written informed consent was obtained from the patient for the publication of this case presentation and accompanying images. A copy of the written consent is available for review by the Editor-in-Chief of this journal.

## Abbreviations

CT: computed tomography; S: segment.

## Competing interests

The authors declare that they have no competing interests.

## Authors' contributions

TG wrote the manuscript. TG and RK performed surgery. AM carried out the pathological examination. RK was involved in the final editing. All authors approved the final manuscript.
